# Prevalence of potential mediators of artemisinin resistance in African isolates of *Plasmodium falciparum*

**DOI:** 10.1186/s12936-021-03987-6

**Published:** 2021-12-02

**Authors:** Afolabi Owoloye, Michael Olufemi, Emmanuel T. Idowu, Kolapo M. Oyebola

**Affiliations:** 1 Genomic Research in Biomedicine Laboratory, Biochemistry and Nutrition Department, Nigerian Institute of Medical Research, Lagos, Nigeria; 2 Parasitology and Bioinformatics Unit, Department of Zoology, Faculty of Science, University of Lagos, Lagos, Nigeria; 3 Sickle Cell Branch, National Heart Lung and Blood Institute, US National Institutes of Health, Bethesda, MD USA

**Keywords:** Artemisinin-based combination therapy, Partial resistance, *Plasmodium falciparum*, Kelch-13, *Pfcoronin*, *pfatpase6*, Mutations, Africa

## Abstract

**Background:**

The devastating public health impact of malaria has prompted the need for effective interventions. Malaria control gained traction after the introduction of artemisinin-based combination therapy (ACT). However, the emergence of artemisinin (ART) partial resistance in Southeast Asia and emerging reports of delayed parasite sensitivity to ACT in African parasites signal a gradual trend towards treatment failure. Monitoring the prevalence of mutations associated with artemisinin resistance in African populations is necessary to stop resistance in its tracks. Mutations in *Plasmodium falciparum* genes *pfk13*, *pfcoronin* and *pfatpase6* have been linked with ART partial resistance.

**Methods:**

Findings from published research articles on the prevalence of *pfk13*, *pfcoronin* and *pfatpase6* polymorphisms in Africa were collated. PubMed, Embase and Google Scholar were searched for relevant articles reporting polymorphisms in these genes across Africa from 2014 to August 2021, for *pfk13* and *pfcoronin.* For *pfatpase6*, relevant articles between 2003 and August 2021 were retrieved.

**Results:**

Eighty-seven studies passed the inclusion criteria for this analysis and reported 742 single nucleotide polymorphisms in 37,864 *P. falciparum* isolates from 29 African countries. Five validated-*pfk13* partial resistance markers were identified in Africa: R561H in Rwanda and Tanzania, M476I in Tanzania, F446I in Mali, C580Y in Ghana, and P553L in an Angolan isolate. In Tanzania, three (L263E, E431K, S769N) of the four mutations (L263E, E431K, A623E, S769N) in *pfatpase6* gene associated with high in vitro IC_50_ were reported. *pfcoronin* polymorphisms were reported in Senegal, Gabon, Ghana, Kenya, and Congo, with P76S being the most prevalent mutation.

**Conclusions:**

This meta-analysis provides an overview of the prevalence and widespread distribution of *pfk13, pfcoronin and pfatpase6* mutations in Africa. Understanding the phenotypic consequences of these mutations can provide information on the efficacy status of artemisinin-based treatment of malaria across the continent.

**Graphical Abstract:**

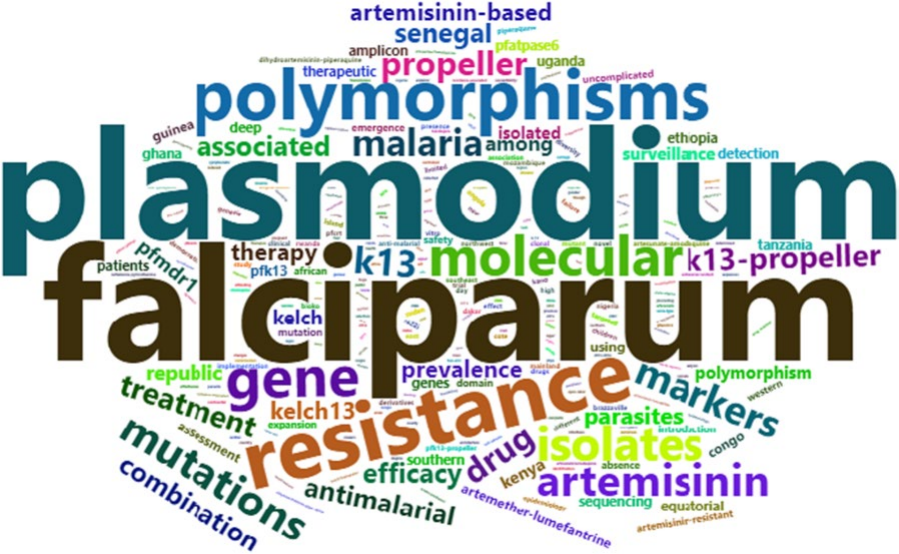

**Supplementary Information:**

The online version contains supplementary material available at 10.1186/s12936-021-03987-6.

## Background

Malaria is a leading cause of mortality and morbidity especially among children under five years old in Africa [1]. Interventions such as insecticide-treated nets, space spraying, indoor residual spraying, larval control, and anti-malarial therapeutics have been adopted to reduce malaria burden across the continent [2]. However, these strategies can be limited by the emergence of resistant strains of both mosquitoes and parasites [3–5].

A major setback to malaria control is the emergence and spread of partial resistance to artemisinin, defined as slow parasite clearance expressed in early ring-stage parasites and polymorphisms in the parasite Kelch-13 propeller gene [6–10]. *Plasmodium falciparum* Kelch-13 (*pfk13*) encodes a protein with 726 amino acids. The polypeptide consists of a poorly conserved N-terminal region (*Apicomplexa*-specific; amino acid from 1 to 211) and three highly conserved regions [10]. It has a coiled-coil-containing ((CCC); amino acid from 212 to 341), broad-complex, tram track, and bric-a-brac ((BTB); amino acid from 350 to 437) and a C-terminal kelch-repeat propeller ((KREP); amino acid from 443 to 726), which harbours virtually all *pfk13* allelic variants associated with artemisinin resistance [11]. Kelch-13 gene is putatively associated with intra-erythrocytic growth and proliferation of *P. falciparum* asexual parasites [12, 13]. Validated *pfk13* gene mutations that have been associated with partial artemisinin resistance include C580Y, R561H, F446I, P574L, N458Y, I543T M476I, R539T, Y493H, and P553L [1, 10, 14].

In addition, polymorphisms in *P. falciparum* coronin (*pfcoronin*) gene have been linked with possible artemisinin resistance [15, 16]. The *pfcoronin* protein belongs to the actin-binding protein family, which has been associated with the motility of sporozoites [17]. *Pfcoronin* encodes a protein with 602 amino acids. The propeller domain of *pfcoronin* has seven blades. This domain is made up of the WD40 repeats (tryptophan-aspartic acid 40) and a β-propeller in the N-terminus region [18]. *Pfcoronin* is involved in organization of F-actin via its N-terminal propeller region and localizes to the parasite membrane [17]. Demas et al. [16] demonstrated in long-term cultivation of Senegalese isolates that G50E, R100K and E107V polymorphisms in *pfcoronin* reduced the susceptibility of the parasites to dihydroartemisinin, the active component of artemisinin. However, these three *pfcoronin* mutations were not detected in field isolates obtained from various endemic countries in Africa, instead a new mutation P76S has been identified [19–21]. This P76S mutation, however, has not shown any predictive effect on reduced efficacy to artemisinin derivatives [19, 20]. As the *pfcoronin* gene is structurally similar to the six-bladed *Pf*Kelch13 propeller domain [18], the extent of interaction between *pfcoronin* and *Pf*kelch13 mutations requires further examination [15].

Previous studies investigated the association *pfatpase6* mutations and artemisinin resistance [22, 23]. The theory of association was based on the mechanism of action of artemisinin on *pfatpase6*, which aggravates calcium homeostasis of *P. falciparum* [24, 25]. Sarco-endoplasmic reticulum Ca^2+^ATPase (SERCA), the calcium pump of sarcoplasmic reticulum responsible for refilling calcium in the endoplasmic reticulum (ER) stores, is critically important for cellular homeostasis and calcium transport and signalling functions [26, 27]. Dissimilar from vertebrates possessing three SERCA genes [28], *P. falciparum* has a single SERCA gene, otherwise known as *pfatpase6* [29]. While some *pfatpase6* polymorphisms have been reported in field isolates [30], the consensus is that *pfatpase6* is not directly involved in artemisinin action or resistance [12, 31]. Be that as it may, the current reliance on artemisinin derivatives for falciparum malaria treatment has stressed the importance of a synergistic effort to monitor the emergence and spread of mutations linked with artemisinin resistance in Africa. This meta-analysis collated the prevalence of *pfk13*, *pfcoronin* and *pfatpase6* polymorphisms across different endemic settings in Africa.

## Methods

This article followed the guidelines for systematic reviews and meta-analyses [32]. Published research documents and collated data on the prevalence of *pfk13*, *pfcoronin* and *pfatpase6* mutations across endemic countries in Africa were used in this report. Two electronic biomedical databases (PubMed and Embase) were methodically explored for peer-reviewed *pfk13* and *pfcoronin* articles published between 2014 and 2021, and for *pfatpase6*, peer-reviewed articles published between 2003 and 2021, which had the relevant study populations (i.e., clinical or community surveys), study design and expected outcomes for this review. Google Scholar was also combed for relevant peer-reviewed articles. Both interventional and observational studies were retrieved and included in the review using the “MeSH” search terms “OR” and “AND’’: “kelch13” OR “kelch-13” OR “*pfk13*” OR “*Pf*kelch13” OR “*Pf*kelch-13” OR “*Plasmodium falciparum* drug resistance” OR “ATP6” OR ““*Plasmodium falciparum* ATP6” OR “*Plasmodium falciparum* ATPase6” OR “*Pf*ATP6” “*pfatpase6*” OR “*Plasmodium falciparum* coronin” OR “*pfcoronin*” OR “*Plasmodium falciparum* coronin” OR “molecular marker” OR “*Plasmodium falciparum*” “*P. falciparum*” OR “falciparum malaria” AND (“African” OR “Africa” OR with each name of the 54 countries in Africa). The citations of the individual search were saved and sent to the reference manager (EndNote version 9.0). The full texts of retrieved citations were downloaded using EndNote. Articles with data from unknown countries and/or sampling sites as well as systematic reviews, conference presentations, letters or correspondence to editors and abstracts with insufficient information were removed.

### Inclusion criteria

The articles included in this review strictly reported *P. falciparum* artemisinin resistance markers, single nucleotide polymorphisms (SNPs) in African countries, polymorphisms in *pfk13*, *pfcoronin* and/or *pfatpase6* confirmed through targeted or whole-genome sequencing. Articles written in English language, from cross-sectional studies such as clinical or community surveys were included, in addition to longitudinal studies of treatment efficacy. Specific studies reporting synonymous and non-synonymous SNPs in *pfk13*, *pfatpase6* and *pfcoronin* were eligible for this meta-analysis.

### Exclusion criteria

Articles reporting molecular markers other than *pfk13*, *pfatpase6*, and *pfcoronin* were excluded from this review. In addition, studies with no definite *pfk13*, *pfatpase6*, and *pfcoronin* SNPs reported either in the main manuscript or Additional file 1 were excluded. Studies reporting *pfk13*, *pfatpase6*, and *pfcoronin* polymorphisms without sequencing techniques were not included.

### Definitions

Partial resistance to artemisinin refers to delayed or slow clearance of ring-stage malaria parasites from the bloodstream following treatment with an artemisinin-based combination therapy [27]. Delayed parasite clearance does not necessarily cause treatment failure. This review utilized the World Health Organisation list of *P. falciparum* artemisinin resistance SNPs classifying *pfk13* mutations into validated and candidate SNPs [33]. *Pfk13*-validated SNPs are significantly associated with reduced drug susceptibility in laboratory assays and a slow parasite clearance rate in field studies [33]. Validated-*pfk13* SNPs include C580Y, R561H, F446I, P574L, N458Y, I543T M476I, R539T, P553L, and Y493H [14]. On the other hand, candidate SNPs are mutations associated with slow parasite clearance in clinical trials but not confirmed in vitro [33]. These include P441L, G449A, C469F/Y, A481V/C, R515K, P527H, N537I/D, G538V, V568G, R622I, and A675V [14]. Other rare variants reported to be associated with delayed clearance but at low frequencies include D452E, C469Y/W, K479I, R515K, S522C, N537D, R575K, M579I, D584V, P667T, and H719N [14].

### Data extraction

**Fig. 1:**
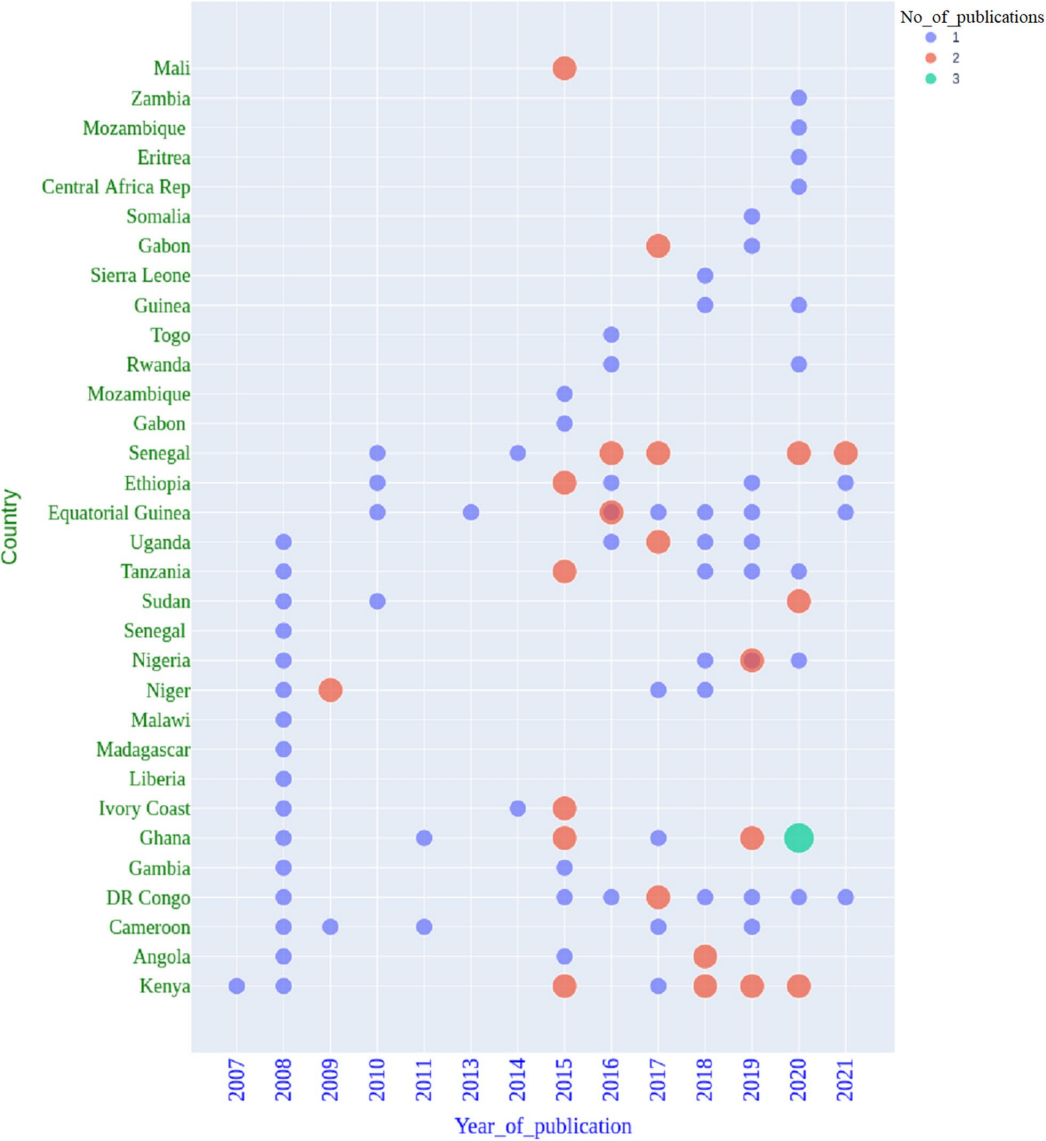
Year of publication of research articles reporting *Pf*k13, *Pf*coronin and *Pf*ATPase6 gene mutations. The coloured dots represent the number of publications reporting polymorphisms in the *Pf*k13, *Pf*coronin and *Pf*ATPase6 genes

**Fig. 2:**
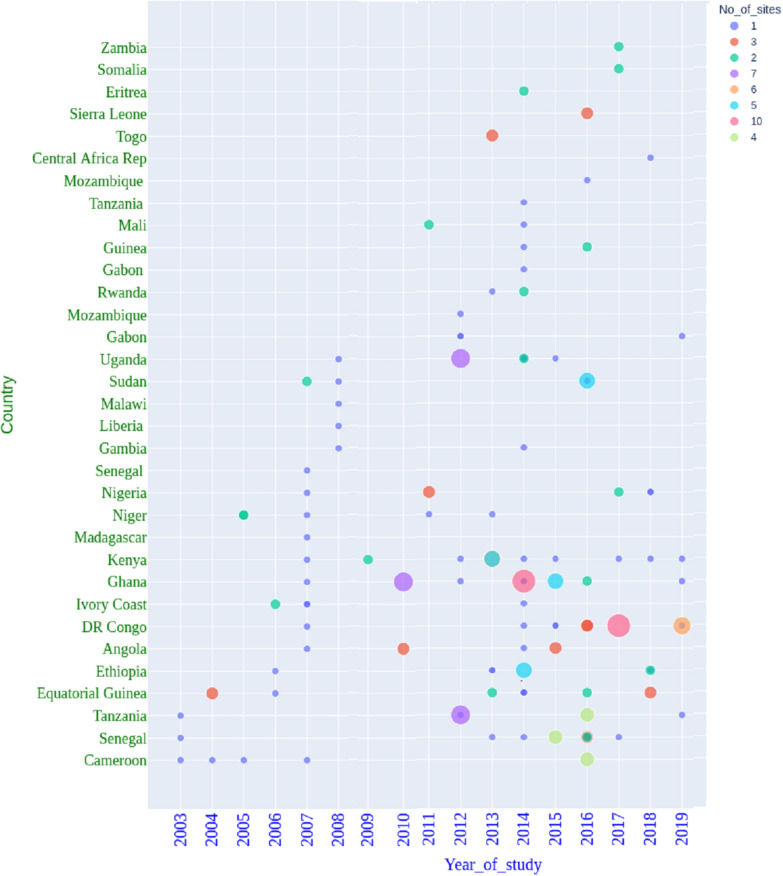
Year of molecular surveillance for *Pf*k13, *Pf*coronin and *Pf*ATPase6 gene mutations. The coloured dots represent the number of sites included in the *Pf*k13, *Pf*coronin and *Pf*ATPase6 studies

**Fig. 3:**
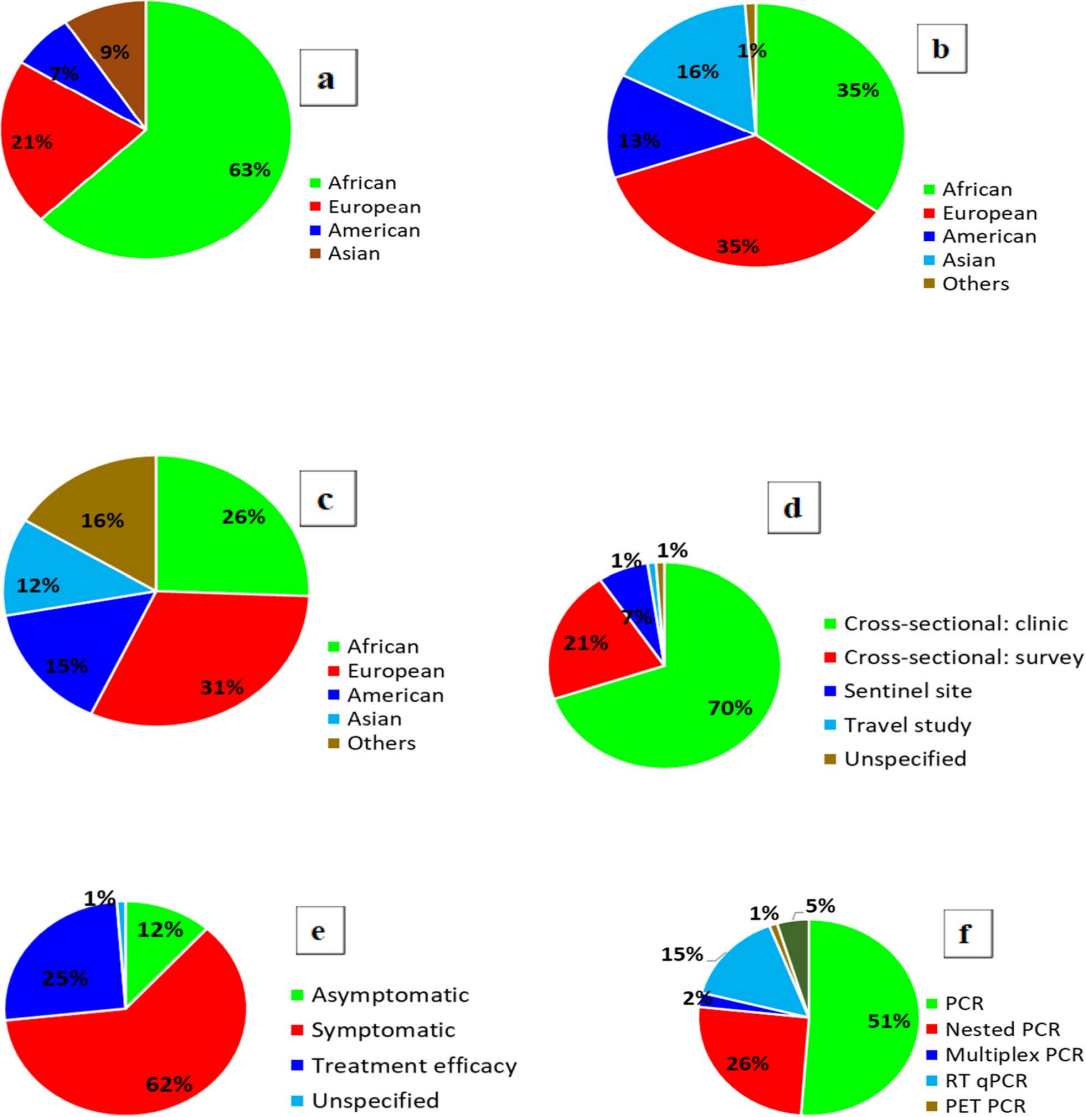
Demography of research articles reporting *Pf*k13, *Pf*coronin and *Pf*ATPase6 gene mutations. **a** First author affiliation, **b** Last author affiliation, **c** Where research funding came from **d** Sampling technique, **e** Clinical status, **f** Parasite detection and/or quantitation assays (PCR = polymerase chain reaction; RT-qPCR = real-time quantitative PCR; PET = photo-induced electron transfer)

The extracted data from each article captured first and last author affiliations, the year the studies were conducted (Fig. 1), year of article publication (Fig. 2), geographic location of the study area, duration of the study, age of the participants and the type of study design (that is, interventional *vs* observational). Data involving sampling strategies, molecular assays performed, clinical status of the study population, and publication affiliation were also reported (Fig. 3).

## Results

**Fig. 4:**
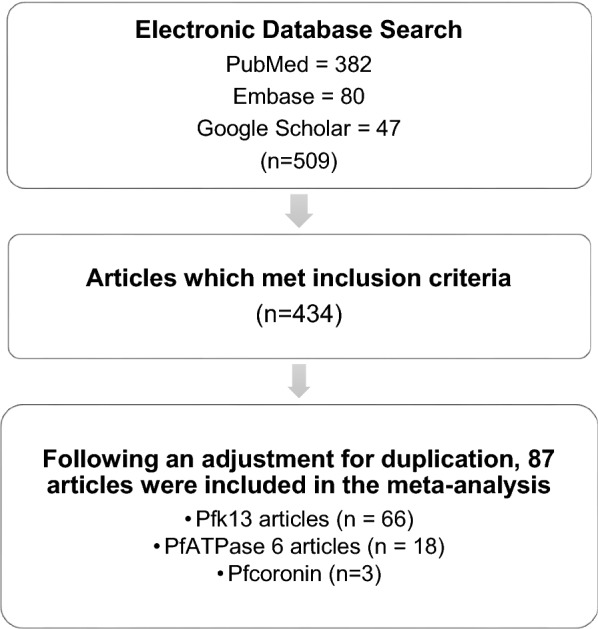
Study selection criteria

PubMed, Embase and Google Scholar databases were combed for relevant articles. The search yielded a total of 509 articles on *pfk13*, *pfcoronin* and *pfatpase6* SNPs, of which 434 articles met inclusion criteria (Fig. 4). Eleven articles with unobtainable full texts were removed. Following an adjustment for duplication (i.e., research articles from the same authors which gave multiple search results or were probably pre-printed before publication), redundant articles were discarded. A total of 87 studies (66 on *pfk13*, three on *pfcoronin* and 18 on *pfatpase* 6) analysed 37,864 (33,383, 1,498 and 2,983) isolates for *pfk13*, *pfcoronin* and *pfatpase* 6 polymorphisms, respectively. The isolates were collected in 29 African countries.

### Sample pre-processing and pfk13 genotyping

The majority of the studies collected blood samples for genotyping on filter paper [34–38] while others did not report the method used for collection [21, 39]. *Plasmodium falciparum* polymerase chain reaction positive (PCR +) samples were 18,292 out of 32,406 total samples collected [39–45], yielding PCR positivity rate of 56.4% malaria in both clinical and community studies. However, five studies did not report the number of PCR + samples [46–50]. *pfk13* gene was successfully genotyped in 15,861 (86.71%) samples using techniques such as targeted and whole-genome sequencing [35, 46–48]. The variant-calling algorithms and data analysis software used included Mega software, Jalview, Phylo, DnaSp, Genescan, Genome Assembly Program, PROVEAN and RStudio [51–54].

### Prevalence of pfk13 non‑synonymous mutations across Africa

**Fig. 5:**
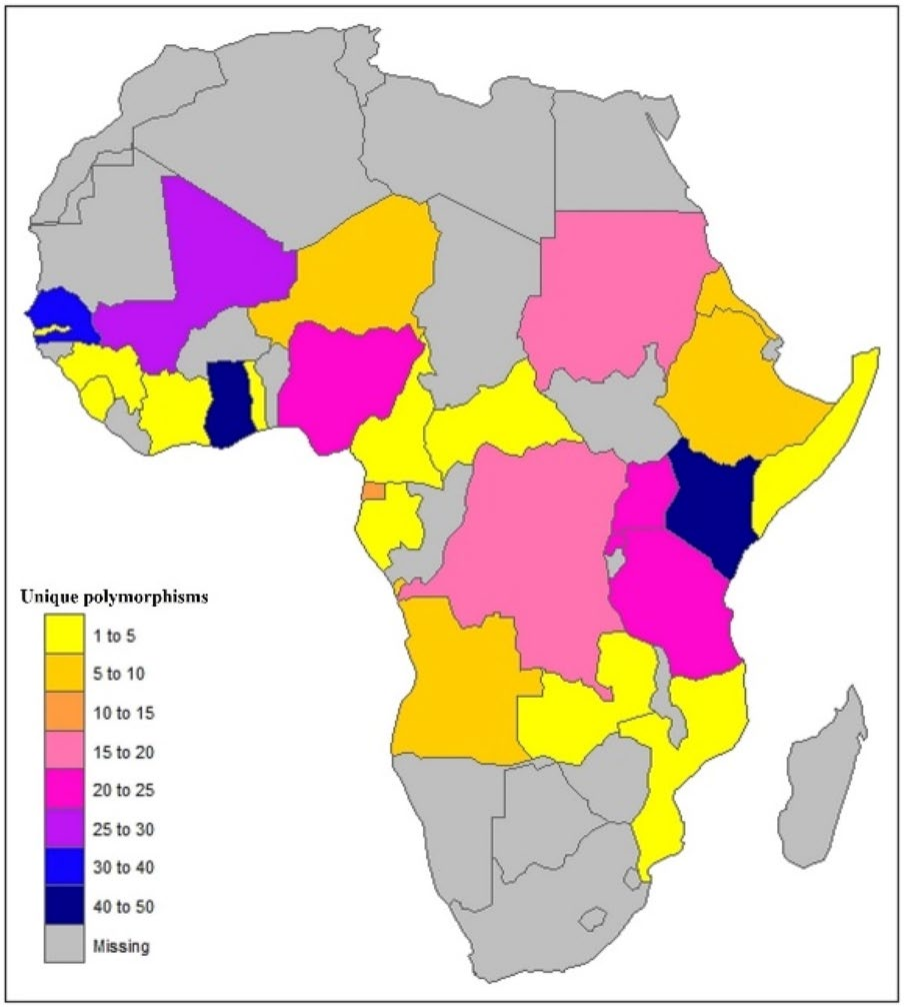
Distribution of unique non-synonymous *P. falciparum* Kelch-13 single nucleotide polymorphisms (SNPs) in African countries [34–46, 48–56, 59, 60, 62–64, 66–68, 73, 74]

At least one non-synonymous *pfk13* mutation was observed in 26 African countries (Fig. 5). The reported *pfk13* non-synonymous SNPs occurring inside the propeller domain (amino acid from 443) include A578S/D/V (95 parasite isolates with the SNP); R561H (20 isolates with the SNP); R622G/K/I (20 isolates with SNP); N587K/I (16 isolates); V555A/L (9 isolates); S522C/M/N (9 isolates); T677A/K/R (9 isolates); Q613E/H (7 isolates), F509G (7 isolates) and V637I (6 isolates); N554H/K/D and A626S/T/V (5 isolates each); and, N609D/L/S (5 isolates) [35, 55–58]. The most frequently reported mutations outside the propeller domain (amino acid below 443) include K189T/N (105 isolates with the mutation) [51, 59], E208K (10 isolates) [60], N142NN (9 isolates) [61], T149S (6 isolates) [62], E433D (4 isolates) [54], and E401Q. Apart from D389H/N/Y (3 isolates), K378R (2 isolates) and D281V (2 isolates), other reported mutations outside the propeller domain (31/40) occurred singly [54, 59]. K189T/N mutation had a high prevalence in Senegal [51, 61]. Ten validated-*pfk13* mutations (C580Y, R561H, F446I, P574L, N458Y, I543T M476I, R539T, Y493H, P553L) have been associated with artemisinin partial clearance [14] of which three (R561H, P553L/T, M476I) were identified in Africa [39, 55, 59]. R561H was identified in Rwanda and Tanzania [39, 63], P553T in Senegal [59], P553L in a patient returning from Angola to China [64], and M476I in Tanzania [55]. In two isolates from Ghana [40], asparagine in position 458 (N458) was found to be replaced by aspartic acid (D) instead of tyrosine (Y).

### Prevalence of pfatpase6 and pfcoronin non‑synonymous mutations in Africa

Six studies reported *pfatpase6* polymorphisms [30, 65–69]. The studies involved 1,323 samples of which 752 *P. falciparum* isolates were PCR + , 644 (85.63%) were successfully sequenced [65–68]. In Tanzania [30], three (L263E, E431K, S769N) of the four mutations (L263E, E431K, A623E, S769N) in *pfatpase6* gene were reported. *Pfatpase6* E431K was reported in Congo and Ethiopia.

**Fig. 6:**
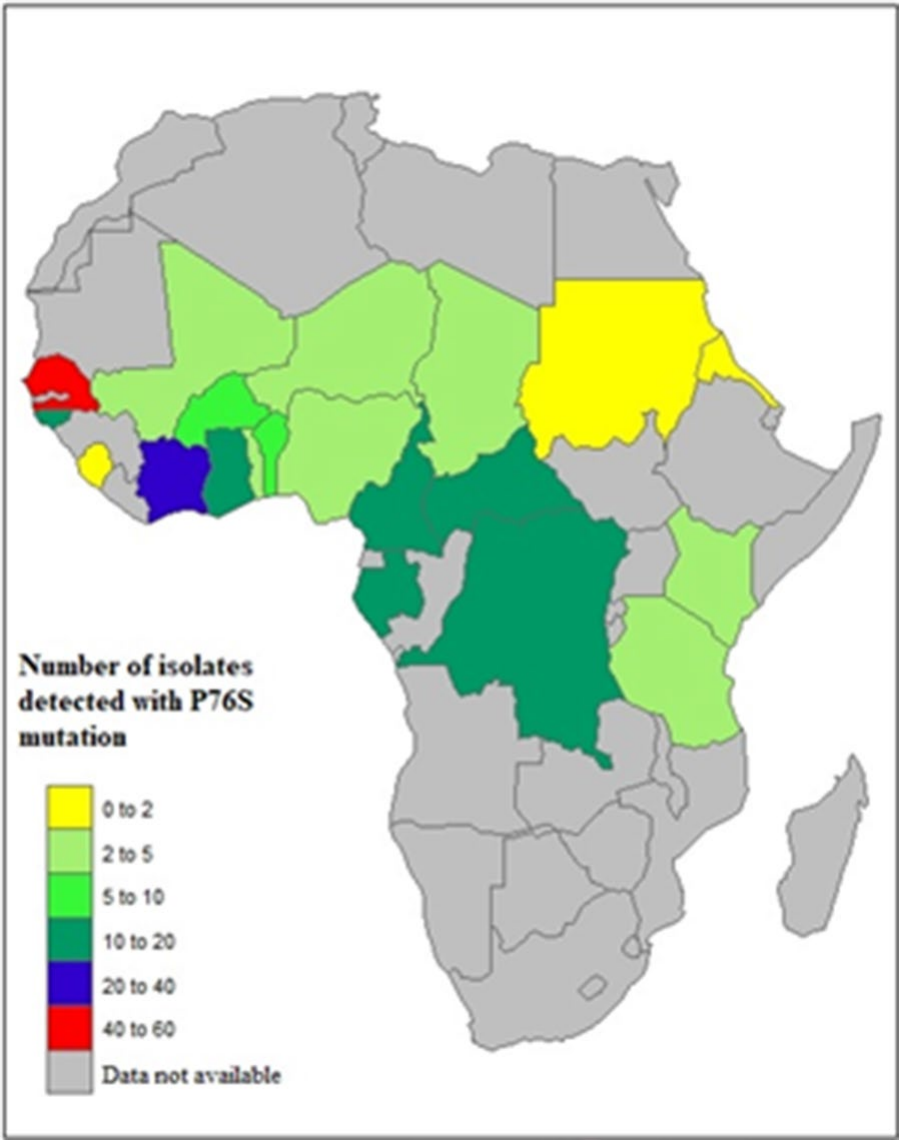
Number of *P. falciparum* isolates with *Pf*coronin P76S mutation in Africa [16, 19, 21]

Three studies reported *pfcoronin* mutations in PCR + field isolates. *Pfcoronin* gene was sequenced in 1,498 (100%) isolates [19–21]. *Pfcoronin* mutations were reported in 21 countries: Ivory Coast, Guinea, Togo, Burkina, Benin, Mali, Nigeria, Senegal, Niger, Ghana, Sierra, Cameroon, Gabon, Democratic Republic of Congo, Central Africa, Chad, Mayotte, Eritrea, Tanzania, Sudan, and Kenya (Fig. 6); P76S polymorphism was identified in all 21 countries [19–21]. The frequency of P76S was higher in Senegal compared to the other countries (Fig. 6). V62M was reported in Ghana, Burkina Faso, Nigeria, Cameroon, Central Africa Republic, Chad, and Gabon (Table 1, Fig. 6). *Pfcoronin* polymorphism was associated with reduced susceptibility in *P. falciparum* adapted long-term to artemisinin [16].

**Table 1:** The prevalence of *Pf*coronin single nucleotide polymorphisms in Africa

Country	Mutation (no of isolates with mutation)	Prevalence of P76S (%)	References
Senegal	P76S (54)	26.3	[19, 20]
Ivory coast	P76S (34)	16.6	[19]
Cameroon	P76S (19), V62M (2)	9.3	[19]
Gabon	V62M (7), P76S (16)	7.8	[19, 21]
Ghana	I53I (6), V62M (6), K69I/R (3), P76S (11), N110Y (6)	5.4	[19, 21]
Democratic Republic of Congo	K69I/R (11), P76S (11), N110Y/D (5), N112Y (10, K115E (1), L121F (6), K127E (6), K127I/R (5), N134Y/D (4), N137I/Y/D (12),	5.4	[19, 21]
Guinea	P76S (11)	4.9	[19]
Central Africa Republic	P76S (10), V62M (2)	4.9	[19]
Burkina Faso	P76S (6), V62M (2)	2.9	[19]
Benin Republic	P76S (6)	2.9	[19]
Mali	P76S (4)	2.0	[19]
Nigeria	P76S(4), V62M (1)	2.0	[19]
Kenya	P76S (4)	2.0	[19, 21]
Togo	P76S (3)	1.5	[19]
Niger	P76S (3)	1.5	[19]
Chad	P76S (3), V62M (2)	1.5	[19]
Mayotte	P76S (2)	1.0	[19]
Tanzania	P76S (2)	1.0	[19]
Sierra	P76S (6)	0.5	[19]
Eritrea	P76S (6)	0.5	[19]
Sudan	P76S (6)	0.5	[19]

## Discussion

The emergence of partial resistance to artemisinin in Southeast Asia (SEA) is an imminent danger to successful malaria control and elimination. The broad spectrum of polymorphisms in genes implicated in artemisinin resistance reported so far in Africa raises concern about potential adaptation of *P. falciparum* to artemisinin. Although the efficacy of current therapy remains high on the continent, there are emerging indications of varying parasite clearance times [70, 71].

Compared to SEA, low prevalence of *pfk13* polymorphisms was recorded across Africa. This could be associated with the later introduction of artemisinin in Africa (between 2000 and 2005) accompanied by a shorter period of artemisinin drug pressure, unlike in East Asia which experienced early adoption of artemisinin in the 1970s [72]. Reports from SEA identified 10 validated-*pfk13* polymorphisms [14], some of which have been identified in African isolates. For instance, R561H was identified in Rwanda and Tanzania [39] and M476I was detected in Tanzania [55]. Although R539T was not found, R539I was reported in Senegal, and in Kenya R539K was observed [48, 59]. P553T (threonine replaced leucine) was reported in Senegal [59]. The presence of these validated mutations in Africa is a red flag as this could be a precursor to total artemisinin resistance and/or increased selection pressure on partner drugs.

Seven of the 11 candidate *pfk13* mutations [14] have been found in Africa. For instance, V568G and A481C were identified in Kenya [48] and Ghana [40], respectively, while A675V was reported in Kenya, Rwanda and Uganda [50, 73]. The other associated markers identified, including C469W (instead of C469Y) [39, 59, 74], G538S (instead of G538V) [58], G449S/C (instead of G449A) [75], were also found in SEA but their respective amino acid substitutions were different. This raises questions about African parasites with potential artemisinin selection background differing from SEA parasites.

The suspected association of *pfatpase6* and *pfcoronin* with increased IC_50s_ points to the possibility of non-*pfk13* mutations. *Pfatpase6* variants was identified in high frequency in Tanzania [30], although no evidence of delayed parasite clearance in the presence of artemisinin has been established. *Pfatpase6* E431K was reported in Congo, Ethiopia and Ghana [65–67]. The variant was also reported in vitro to be associated with delayed artesunate-treated parasite clearance in Senegal [22]. However, a later study in Iran suggested that the role of E431K variant in artemisinin resistance was suspect [69]. *Pfatpase6* E431K mutation often co-occurs with other *pfatpase6* gene polymorphisms, usually the S769N and L623E mutations [76].

Research on *pfcoronin* as a potential marker of artemisinin resistance in African parasites is relatively recent and still evolving. *Pfcoronin* mutations reported so far include I53I, V62M, K69K/I/R, P76S, N110Y/D, N112Y/D, K115E, L121F, K127E, K127I/R, N134Y/D, N137Y/D, and N137I/S [19–21]. In 21 countries where *pfcoronin* was genotyped, P76S variant was observed in all the populations at varying frequencies: 26.3% in Senegal, 16.6% in Ivory Coast, 9.3% in Cameroon, 7.8% in Gabon, 5.4% in Ghana, 5.4% Democratic Republic of Congo; all other countries at less than 5%. None of the variants suspected to be associated with delayed parasite clearance in the presence of artemisinin pressure (E107V, G50E, and R100K) in laboratory isolates was reported in natural African populations. Even though *pfcoronin* polymorphisms [20, 21] have not yet been validated in clinical isolates as markers of delayed parasite clearance, their structural similarity with *Pf*kelch13 suggests the possibility of a common mechanism of resistance emergence [20, 77]. As much as this meta-analysis is not minutely exhaustive, detailed analysis of the phenotypic effects of reported mutations is recommended to monitor continued efficacy or otherwise of artemisinin-based treatment of malaria in Africa.

## Conclusions

Although artemisinin-based treatment of malaria remains largely potent in Africa and there is no evidence that full resistance has emerged, increased burden of mutations in genes implicated in artemisinin resistance can eventually cause total artemisinin resistance and/or increased selection pressure on partner drugs. This calls for continued therapeutic efficacy monitoring and genomic surveillance across Africa.

## Supplementary Information

**Additional file 1.** Study sites and sample description.

## Data Availability

All data generated or analysed during this study are included in this published article (and its additional files).

## Abbreviations

KREP: Kelch-repeat propeller
PCR: Polymerase chain reaction
*pfatpase6*: *Plasmodium falciparum* ATPase6
*pfcoronin*: *Plasmodium falciparum* Coronin
*pfk13*: *Plasmodium falciparum* Kelch-13
SEA: Southeast Asia
SERCA: Sarco-endoplasmic reticulum Ca2^+^ ATPase
SNP: Single nucleotide polymorphisms
